# Evolution of Microwave Spectroscopy at the National Bureau of Standards (NBS) and the National Institute of Standards and Technology (NIST)

**DOI:** 10.6028/jres.117.016

**Published:** 2012-09-28

**Authors:** F. J. Lovas, D. R. Lide, R. D. Suenram, D. R. Johnson

**Affiliations:** National Institute of Standards and Technology, Gaithersburg, MD 20899

**Keywords:** atmospheric chemistry, dimers, hydrogen bonding, internal rotation, microwave spectroscopy, radio astronomy, rotational spectrum, tunneling motions

## Abstract

This paper describes the beginning and evolution of microwave rotational spectroscopic research starting in 1954 at the National Bureau of Standards (NBS), located at that time in Washington, DC, through the present at NIST in Gaithersburg, MD. David Lide was hired in 1954 to start this research employing Stark modulated waveguide septum cells. When Donald R. Johnson joined the lab in 1968, he developed parallel plate cells coupled with rf and DC discharge methods to study free radicals and transient species. In the mid 1980s Lovas and Suenram constructed a pulsed molecular beam Fourier Transform microwave (FTMW) spectrometer to study hydrogen bonded and van der Waals dimers and trimers. This article describes the types of molecules studied and the type molecular properties derived from these measurements as well as some of the instruments developed for these studies. The two major areas of application described are atmospheric chemistry and molecular radio astronomy.

## 1. Introduction

The first work on microwave spectroscopy at the National Bureau of Standards (NBS) was carried out in the late 1940s by Harold Lyons. This was instigated by NBS Director Edward U. Condon to follow up an idea he got while in his previous position as Director of the Westinghouse Research Laboratories in East Pittsburgh. Westinghouse Labs was the scene of the first high-resolution microwave measurements on record, done by William Good [1] in 1946 on the 23.9 GHz transitions of ammonia. Condon recognized that resonances of this type in low-pressure gases might be used as secondary frequency standards, and might even replace the primary astronomical time/frequency standard in use since the adoption of the Treaty of the Meter in 1875. Lyons succeeded in 1949 in using an NH_3_ microwave line to control a clock. His research showed that the concept was promising, but Doppler and wall-collision broadening limited the line widths that could be obtained, and hence the accuracy of the clock. Rapid advances in atomic and molecular beam measurements soon showed that the beam technique had major advantages over using bulk gases, and the work on the ammonia frequency standard was abandoned.

## 2. Development of Microwave Spectroscopy at NBS

**Fig. 1:**
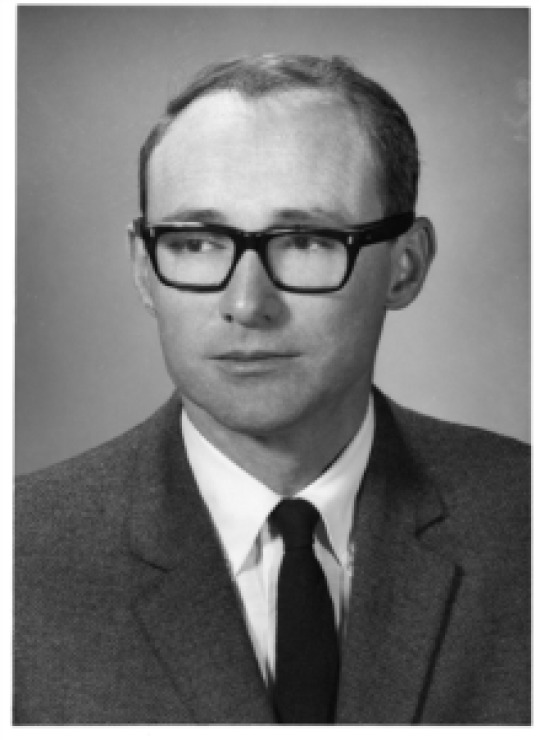
David Lide

NBS began a sustained research program in microwave spectroscopy in 1954 as a part of a major thrust in the fields of thermodynamics and thermophysics. The formulations of statistical mechanics relating macroscopic thermodynamic properties such as heat capacity and entropy to molecular energy levels was well established, and this provided a motivation for investigating vibrational and rotational spectra. The Thermodynamics Section hired David Mann in 1951 to set up an infrared spectroscopy program. Mann had worked with David Lide (see Fig. 1) in the microwave laboratory of E. B. Wilson at Harvard, and in 1954 he recruited Lide to join NBS and start a microwave program. Lab space was obtained and the first equipment ordered in late 1954.

The first spectrometer was a conventional 80 kHz Stark modulation instrument with a 15 foot X-band waveguide cell, very similar to the spectrometers in the Harvard lab. Components that could not be purchased from commercial sources were constructed at NBS or by contractors. The first problem undertaken, serving as a test of instrument, was a reinvestigation of sulfuryl fluoride, SO_2_F_2_ [2], which cleared up a previous error in the dipole moment and provided interesting information on the low-lying vibrational states. A strong Coriolis interaction between the two lowest excited vibrational states was confirmed, and this permitted a determination of the frequency of one of these states, which had not been observed directly in the infrared spectrum. In the future this tool was to be used often to locate vibrational levels that could not be observed directly.

**Fig. 2:**
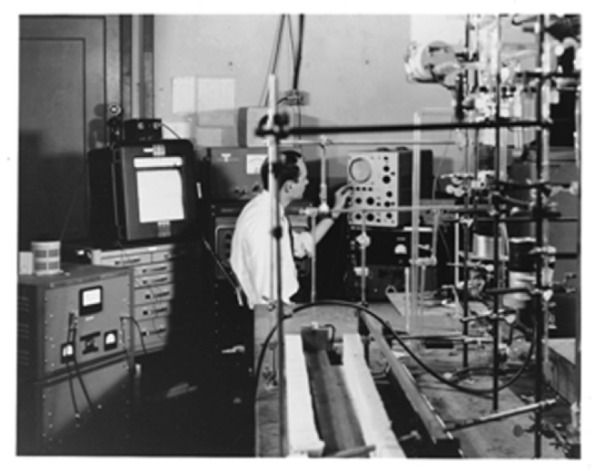
NBS microwave laboratory in 1957

The microwave program initially had two thrusts, internal rotation and other low-frequency motions, and the spectra of transient species. Molecules with internal rotation or inversion modes were characterized by very low-lying energy levels, which made large contributions to the thermodynamic properties. Since thermodynamic data were becoming more important for industrial and military applications, funding could be obtained fairly easily for this work. There was similar interest in the molecular species present in high-temperature systems and plasmas. Lide had first worked on a hindered internal rotation problem while a summer student at the Westinghouse Labs in 1950, where he studied the spectrum of methyl silane and was able to estimate the barrier height from intensity measurements on torsional satellites [3]. In 1956 he started measurements on a series of molecules exhibiting internal rotation with the conventional waveguide spectrometer shown in Fig. 2, while a new spectrometer designed for studying free radicals was under construction.

The permanent staff in the NBS microwave group was limited to David Lide and David Mann during this period, and Mann was concerned mainly with infrared spectra and administrative duties. However, the group was fortunate in being able to bring in a large number of postdoctoral fellows, summer students, and visiting scientists.

This was facilitated by the establishment of the NBS-NRC Postdoctoral program through the efforts of David Mann and Joseph Hilsenrath in the mid-1950s. With the help of this program and others, there was a steady flow of postdocs through the microwave lab. These included, in the 1956–1970 period: Robert Kurland, Victor Laurie, Ted Sarachman, Leonard Nugent, Bill Kirchhoff, Bob Kuczkowski, Ralph Nelson,Wallace Pringle, Don Johnson, Frank Lovas, and Jean Jacob. Several of these postdocs went on to become permanent NBS employees. Foreign Guest Scientists included Takahiro Kasuya and Chi Matsumura from Japan, A. M. Ronn from Israel, and Daniel Christensen from Denmark. Francis Powell from Catholic University and Larry Krisher from the University of Maryland, both local institutions, regularly spent time in the NBS microwave lab over many years. Thus, in spite of tight budgets and NBS personnel restrictions, the microwave group maintained a very active research program.

There were also many interactions with other microwave labs both in the U. S. and abroad. Interactions with Harvard (E. B. Wilson) were particularly strong, but they included other places such as the National Research Council of Canada (C. C. Costain), Columbia (C. H. Townes and B. P. Dailey), Berkeley (R. J. Myers), and Stanford (Victor Laurie). David Lide spent a sabbatical year in 1959–60 with D. J. Millen at University College London and Børge Bak at Copenhagen, and another in 1968 with Paolo Favero at the University of Bologna. Members of the group made many contributions to meetings and conferences, including the Ohio State Molecular Spectroscopy Symposium, American Physical Society and American Chemical Society meetings, the biennial European Molecular Spectroscopy Symposium, and the 1962 International Symposium on Molecular Structure and Spectroscopy in Tokyo.

## 3. Specific Studies from 1954 to the Early 1970s

As mentioned above, the first thrust of the research program at NBS was to study molecules with internal motions that produced low-frequency vibrational modes. These studies are summarized here.

One-top molecules:

- Methyl amine [4,5,6,7]. The complex inversion-internal rotation motions were elucidated, and the torsional barrier height and inversion splitting determined.
- Propylene [8,9] and methylallene [10]. Barrier heights determined.
- Ethyl chloride [11], ethyl bromide [11], and ethyl cyanide [12]. Barrier heights determined.
- Isoprene [13]. Barrier to CH_3_ rotation shown to be higher than propylene and similar molecules.
- Phosphorus trifluoride-borane (PF_3_BH_3_) [14]. A strong Coriolis interaction allowed an accurate determination of the barrier height.
- Trimethylamine-trimethylboron complex [15]. No evidence of internal rotation, but some structural information obtained.

Two-top molecules:

- Propane [16]. The two CH_3_ groups were shown to be staggered, with the CH_3_ axis essentially coincident with the C-C bond. The barrier is high. Propane was shown to have a very small but non-zero dipole moment (0.084 D).
- Isobutene [17]. The equilibrium conformation was determined and the methyl barriers measured.

Three-top molecules:

- Trimethylamine [18], trimethylphosphine [19], and trimethylarsine [20]. The energy levels associated with the torsional motions were determined and limits placed on the barrier heights.
- Isobutane [18,21], tertiary butyl fluoride [19], and tertiary butyl chloride [22]. Each CH_3_ group was found to be hindered by a high barrier, with smaller interactions between the groups.

Low barrier cases:

- Methyltrifluoromethylacetylene [23]. Published measurements reinterpreted to show a near-zero barrier.
- 1-Chloro-2-butyne [24]. Found to have nearly free internal rotation; upper limit to barrier was set.
- Methylsilylacetylene [25]. Barrier was found to be very small but non-zero.
- Trifluoromethyl trifluorosilane (CF_3_SiF_3_) [26]. The barrier to internal rotation was found to be fairly low, leading to a torsional frequency in the far infrared.

Butadiene derivatives:

- Fluoroprene [27] and isoprene [13]: The carbon skeleton was found to be planar with a *tran*s configuration. The barrier to rotation about the C-C bond is very high, with no indication of a *cis* isomer.

Other studies:

- Cyanamide [28,29]. Equilibrium configuration was found to be pyramidal with a very low barrier to inversion.
- Difluoramine (NF_2_H) [30]. Pyramidal configuration with high barrier to inversion.
- Tetrafluorohydrazine (N_2_F_4_) [31]. C_2v_ configuration with very high barrier to internal rotation and inversion.
- Propyl chloride (C_3_H_7_Cl) [32]. Both *trans* and *gauche* conformers were identified and shown to be very close in energy.

The information on internal motions in the molecules listed above proved valuable in gaining an overall picture of hindered internal rotation and in testing various theories and quantum chemical calculations. Furthermore, these studies produced a large amount of precise data on interatomic distances and angles, electric dipole moments, and nuclear quadrupole coupling constants. Other molecules studied in this period for the purpose of measuring structural parameters, dipole moments, and quadrupole coupling constants included:

- Vinyl chloride [33]
- Vinyl fluoride [35]
- Chloroform [37]
- *tert*-Butyl acetylene [39]
- *tert*-Butyl cyanide [39]
- Hexafluoropropene [42]
- *cis*-Difluoroethylene [35]
- Cyclopentene oxide [45]
- Silacyclobutane [47]
- Silacyclopentane [49]
- Hydrazoic acid [34]
- Perchloryl fluoride [35]
- Difluoroborane [38]
- Trifluoramine oxide [40]
- Methyl sulfone [41]
- Methyl sulfonyl fluoride [41]
- Ethynydifluoroborane [43,44]
- Cyanocyclobutane [46]
- 3,6-Dioxabicyclo[3.1.0]hexane [48]

The second major thrust of the NBS microwave lab in the 1960–70 period was directed at the detection and study of molecular species present in highly energetic environments. One motivation for this was the interest at that time in improving rocket performance, searching for new approaches to missile defense, and other military/space program applications. The NBS work involved two techniques, (a) generating short-lived species by electrical discharges and subsequent chemical reactions, then flowing them through a waveguide cell, and (b) producing molecules by vaporizing a solid into an absorption cell maintained at elevated temperature. Successes were achieved with both techniques.

To pursue the first approach, a high-power electrical discharge cell was constructed in which atoms of O, H, etc. could be produced. The gas stream containing these atoms was mixed with another stream containing appropriate molecules and the reaction products pumped through a special absorption cell. The cell [50] was designed for Stark modulation with a Δ*m* = ±1 selection rule. It allowed the gas to flow through freely, and the walls could be coated so as to retard destruction of the transient molecules. This technique was used to study several transient species:

- Sulfur monoxide (SO) [51]. The spectrum of this free radical, an analog of O_2_, is complicated by interaction of the electron spin with the overall rotation. The observed spectrum was explained satisfactorily and the coupling parameters measured.
- Hydroxyl radical (OH) [52]. Although the OH spectrum had been previously observed, the stronger signals obtained with the NBS apparatus allowed the first precise measurement of the electric dipole moment.
- Difluoromethylene (CF_2_) [53,54]. A detailed analysis of the spectrum, including centrifugal distortion effects, led to definitive values of the structure, dipole moment, and vibrational force field.

Refinements and extensions of this technique, to be discussed later, produced important data on a wide range of transient species of interest in radio astronomy and other areas.

The investigations of molecules present in high-temperature gases employed a similar type of waveguide cell, this time contained in a quartz jacket inside a furnace. A tray below the waveguide contained a solid sample of the substance of interest. The molecules studied included:

- Lithium chloride (LiCl) [55]. The rotational constants and interatomic distance were determined with high precision, as well as the variation of dipole moment with vibrational state and the dipole derivative.
- Aluminum monofluoride (AlF) [56,57]. This compound was produced by reacting AlF_3_ with Al in the heated cell. Precise rotational constants, vibration-rotation interaction constants, quadrupole coupling constant, and electric dipole moment were obtained. These measurements indicated a highly ionic Al-F bond.
- Aluminum monochloride [57]. Similar measurements were done on AlCl.
- Cesium hydroxide (CsOH) [58,59,60] and rubidium hydroxide (RbOH) [60,61]. These spectra were observed in the vapor above the solid compound. Interatomic distances, dipole moments, and other parameters were measured, and a rough value of the metal-oxygen stretching frequency was obtained. The molecules were shown to be linear with a very large amplitude bending vibrational mode, which leads to unusual patterns of vibrational satellites.

**Fig. 3:**
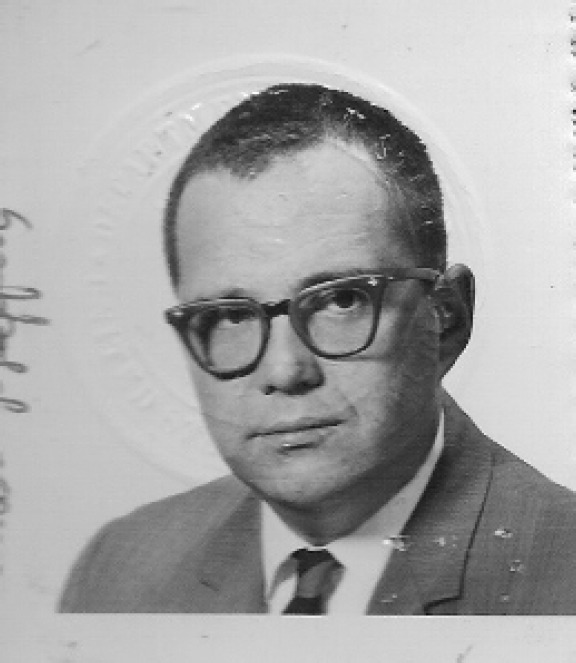
Walter Lafferty

As a result of reorganizations at the National Bureau of Standards in the early 1960s, the microwave group was merged with the high-resolution infrared group that had been built up by Earle K. Plyler. The combined section, called Molecular Spectroscopy, was led by David Lide. Plyler had constructed what was probably the most advanced high-resolution infrared instrument in the world at that time and had brought in several young spectroscopists including Arthur Maki, Walter Lafferty (shown in Fig. 3), and Bruce Olson. This merger led to highly productive symbiosis between the microwave and infrared laboratories. A number of molecules were investigated in both spectral regions, yielding new information on vibration-rotation interactions.

Examples include:

- HCN and DCN [62,63,64,65]
- FCN [66]
- ClCN [67]
- N_2_O [68]
- HCCCN [69]
- CH_3_CN [69]
- DCCD [70]
- OCS [71,72]
- OCSe [73]

**Fig. 4:**
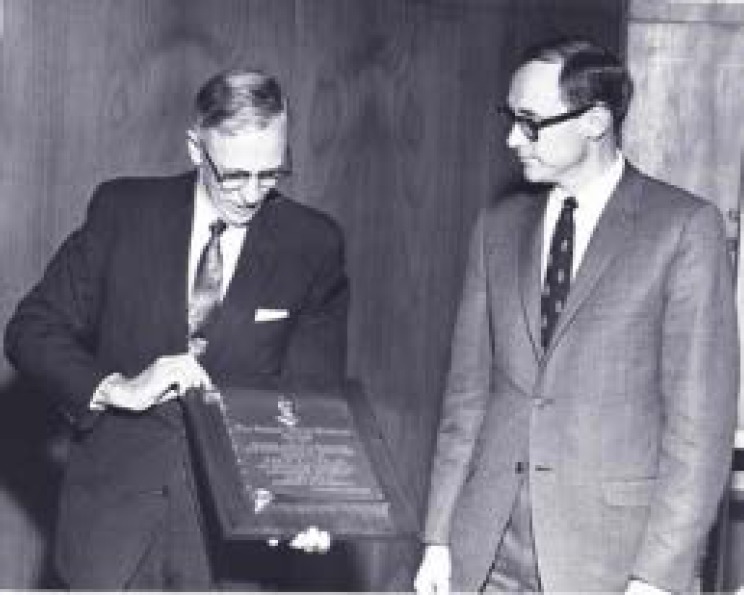
Allen Astin, NBS Directory, presenting the Stratton Award to David Lide in 1968

The work on HCN was notable in that it was used to explain the mechanism of far infrared laser action in HCN, which had been incorrectly attributed to the CN molecule [62,63]. This work led to other measurements that shed light on other gas phase laser transitions such as CS_2_ [74] and DCN and other HCN laser transitions [64,65]. The HCN laser played a significant part in the 1972 measurement of the speed of light to unprecedented accuracy, a measurement that led to a new definition of the meter as the standard of length. In 1968 Lide received the NBS Stratton Award (see Fig. 4) with the citation: “For outstanding research and distinguished authorship in the field of microwave spectroscopy”.

**Fig. 5:**
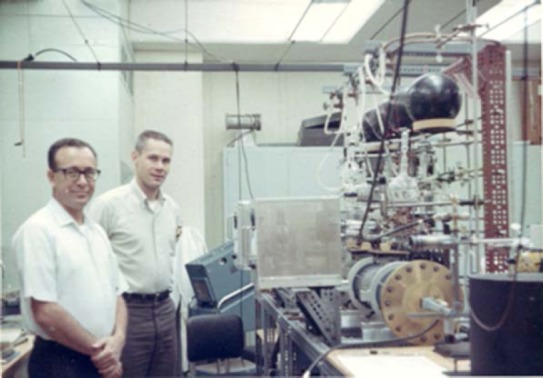
Francis X. Powell (left) and Donald Johnson in new laboratory space at the Gaithersburg, MD site around 1969

In the fall of 1967, Don Johnson, from the C. C. Lin group at the University of Oklahoma, joined the microwave team at NBS as a Postdoctoral fellow under the guidance of David Lide. New laboratory space for the Molecular Spectroscopy Group had just been completed at the Gaithersburg site. Working with Francis X. Powell, a guest researcher from Catholic University, Johnson’s first task was to set the equipment from the Connecticut and Van Ness site in the new laboratory space in Gaithersburg to provide for future research (see Fig. 5). It had been decided that the focus of much of the future research effort in the new laboratory would be on developing microwave techniques for studying short lived chemical intermediates that play important roles in many gas phase reactions. Fortunately, the Molecular Spectroscopy group had amassed huge collection of spectroscopic equipment from past experiments. Perhaps the most important to the future efforts was an amazing collection of reflex klystrons providing tunable radiation from about 2 GHz to well over 100 GHz.

The technology of microwave spectroscopy was still at a very primitive stage in 1967 and most of the apparatus that would be needed had to be designed and built for the task. Most of the short lived molecules that Powell and Johnson hoped to study had not been studied in the gas phase before and their microwave spectra could not be predicted very well. They could anticipate lengthy searches for very weak signals so the microwave sources they intended to use needed to be stabilized both electronically and thermally while remaining easy to use on a daily basis. Commercial electronic stabilization equipment was just being developed at the time and was too difficult to use for broad searches requiring several different klystrons. Their solution was very simple. Each klystron was sealed and submerged in its own 5 gallon container of automotive motor oil and operated with reduced dc voltage on the filament. They also developed their own vacuum tube sweep circuits and signal amplifiers in order to keep the system noise as low as possible.

## 4. Applications in Radio Astronomy and Atmospheric Chemistry

**Fig. 6:**
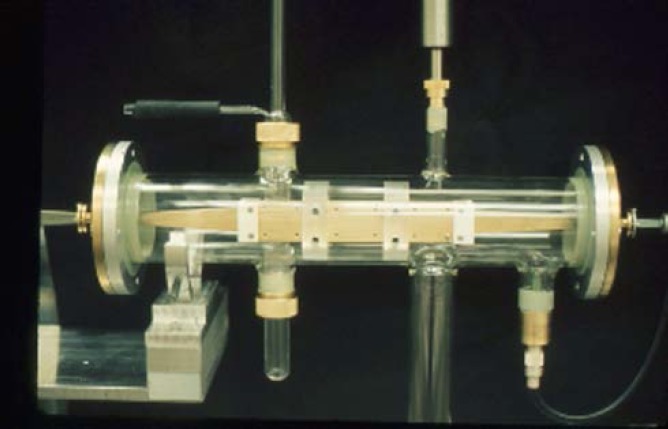
Parallel plate millimeter wave Stark cell

**Fig. 7:**
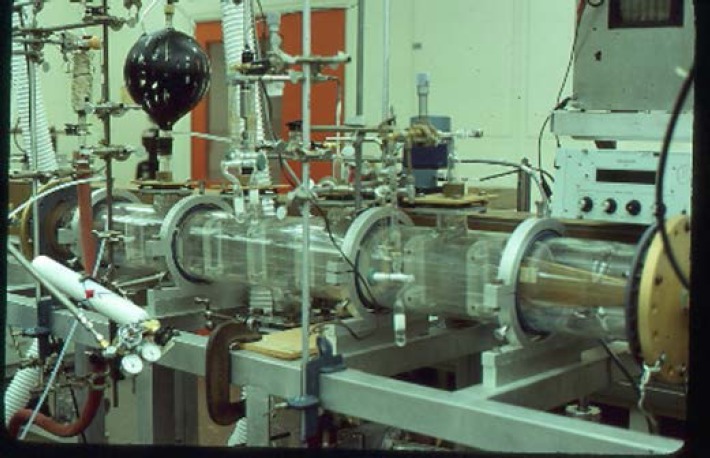
Centimeter wave parallel plate Stark cell

Most of the short lived molecules that Johnson and Powell hoped to study would be likely to exist only in such small concentrations that their absorption path length would be a few centimeters at best. The parallel plate absorption cells shown in Fig. 6 and Fig. 7 were developed for these studies with Teflon coated plates and very high speed pumping systems to allow the chemistry to be optimized in electrical discharge production systems. The free radical, ClO, was the first to be studied [75] with this system followed quickly by BrO [76] and SF_2_ [77]. In later years ClO and BrO became quite important in the atmospheric reaction mechanisms in the destruction of the ozone layer.

In 1969 the first organic interstellar molecule was detected by L.E. Snyder, and coworkers [78]. This inspired Johnson to undertake the study of a related molecule, thioformaldehyde (H_2_CS) [79] which was detected in interstellar clouds a few years later towards Sgr B2 at the Parkes 64 m radio telescope in Australia [80].

In 1970 Frank Lovas joined the NBS microwave group as a Postdoctoral fellow of David Lide. Since Lide had left the group in 1969 to direct the Office of Standard Reference Data, Lovas and Johnson began collaborating on lab studies. Their first joint study was on the radical, BF, [81] produced in a microwave discharge of BF_3_. Later, they studied the transient molecule CH_2_=NH [82] produced by fluorine atom abstraction from methyl amine. These experiments employed the millimeter wave parallel plate cell shown here. CH_2_NH is isoelectronic with formaldehyde and thus a potential interstellar molecule. As in the case of thioformaldehyde, an Australian team using the Parkes 64 m radio telescope detected the methanimine 1_10_ – 1_11_ transition with ^14^N hyperfine structure toward the galactic center cloud Sgr B2 [83].

These early laboratory studies on interstellar molecules pointed to the need of comprehensive microwave spectral data, i.e. spectral predictions beyond the measured data sets, so Johnson, Lovas and Kirchhoff initiated the data series “Microwave Spectra of Molecules of Astrophysical Interest” with the first publication on Formaldehyde, Formamide and Thioformaldehyde [84] with predicted spectra up to 300 GHz which covered the receiver range of radio telescopes at that time. Kirchhoff had developed the fitting code for other projects but it was ideal for predicting spectra with firm statistical uncertainties [85].

In 1972 Bill Kirchhoff left the Molecular Spectroscopy Section and joined the Office of Air and Water Measurements, thus providing an opening to hire Lovas into the Molecular Spectroscopy Section. In addition to continuing microwave lab studies, one of Lovas’ duties was to take over the Molecular Spectroscopy Data Center funded by the Office of Standard Reference Data. This involved the production of the Microwave Spectral Tables, the first on Diatomic molecules [86] and continuing the series on Microwave Spectra of Molecules of Astrophysical Interest. In 1973–74 Eberhard Tiemann from the Freie Universität Berlin spent a year sabbatical working with Lovas and Johnson on studies of transient species such as the SO-dimer, OSSO, [87] produced in a microwave discharge of SO_2_ employing the centimeter wave parallel plate cell shown in Fig. 7.

**Fig. 8:**
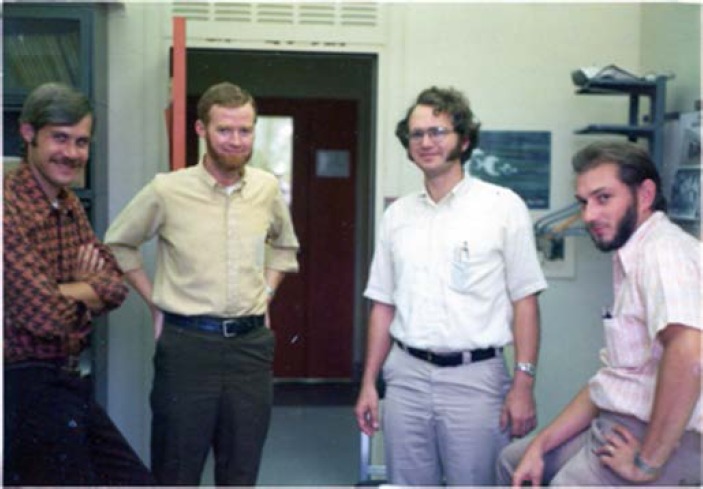
From the left: Don Johnson, Frank Clark, Richard Pearson, and Frank Lovas

**Fig. 9:**
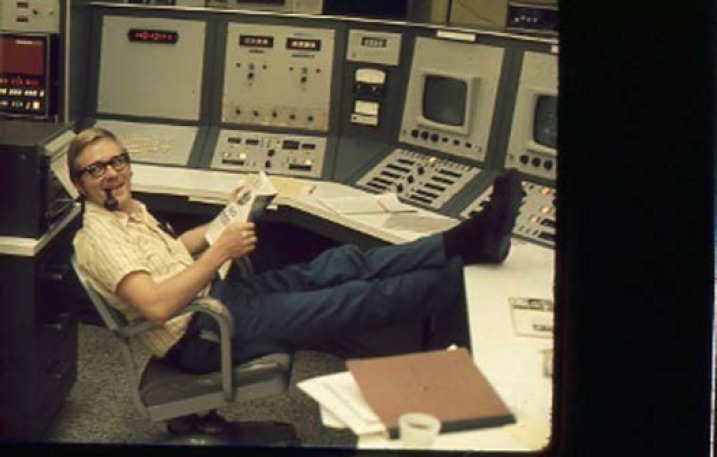
Lew Snyder at the console of the NRAO 140 ft radio telescope at Green Bank, WV

During the early 1970s Johnson and Lovas began a long-term collaboration with Lew Snyder (see Fig. 8) and his students at the University of Illinois with the detection of interstellar dimethyl ether [88] toward the Orion Nebula. Also in 1973 one of Snyder students, F. O. Clark (see Fig. 9) joined NBS as a Postdoctoral fellow with Johnson to carry out both lab and interstellar molecular studies. The first lab study Clark worked on with Lovas and Tiemann was the pyrolysis of ethylamine to produce the transient species vinyl amine [89]. On the interstellar front, Johnson, Clark, and Lovas joined a host of other astronomers in the detection of trans-ethanol toward SgrB2 [90]. Also in 1975 Johnson and Lovas were co-authors of a paper describing the detection of interstellar sulfur dioxide, SO_2_, with L.E. Snyder as lead author [91].

In 1974 Richard Pearson (see Fig. 9) joined the microwave group as a Postdoctoral fellow with Lovas. Pearson was involved in a variety of spectral studies, but one important result was the determination of the structure of CH_2_NH [92]. As opposed to producing CH_2_NH with F atom abstraction from methylamine, Lovas and Pearson employed pyrolysis of methylamine and ^13^C or ^15^N isotopically enriched forms. For the normal species the best production was found to be from pyrolysis of 1,2-diaminoethane (ethylenediamine) as noted in the earlier study of vinyl amine [89].

**Fig. 10:**
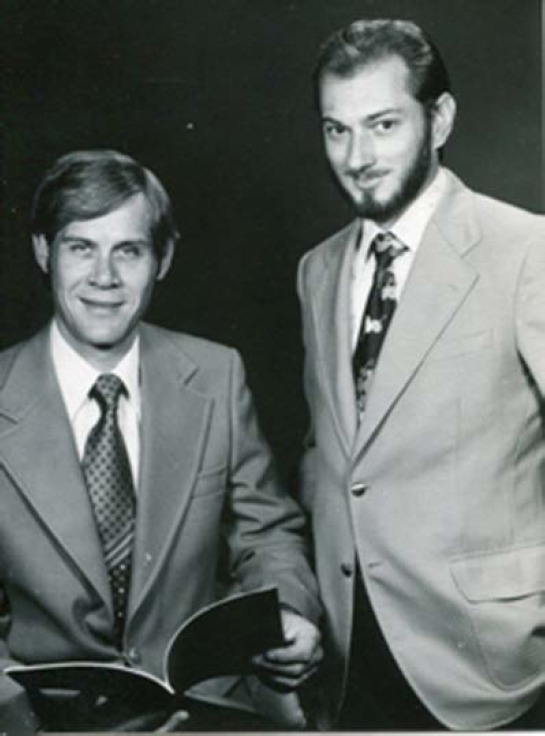
Don Johnson and Frank Lovas after receiving the Department of Commerce Gold Medal Award in 1976

Based on observations of several Harvard astronomers, which were communicated to Johnson and Lovas, ethyl cyanide was identified in the Orion Nebula cloud OMC-1 [93] by means of 24 transitions. The existing laboratory data at that time were measurements below 41 GHz, while the astronomical data set ranged from 88 GHz to 116 GHz, so the literature data was supplemented with new measurements in the 89 GHz to 118 GHz range to provide a firmer identification. As a result of their laboratory and astronomical studies on interstellar molecules, Johnson and Lovas (see Fig. 10) were awarded the Department of Commerce Gold Medal in 1976, the highest award of the Department, with the citation “For their outstanding contributions to the interdisciplinary application of microwave spectroscopic techniques to aeronomy, astronomy, chemistry, and industry”.

In 1975 Rick Suenram joined the group as a Postdoctoral fellow with Don Johnson with an interest in atmospheric and interstellar molecules. One of the first lab studies Suenram worked on with Johnson and Lafferty was the microwave spectrum of cyanamide [94], which had been detected in the Sgr B2 interstellar cloud by Turner et al. [95] the previous year. Suenram and Johnson [96,97] also began a series of studies of the chlorine nitrate molecule (ClONO_2_), which was found to be an important atmospheric species in reactions causing the destruction of the ozone layer. A number of years later, Suenram and Lovas extended the spectral measurements on chlorine nitrate to the millimeter-wave range [98] since it was becoming clear that atmospheric monitoring of chlorine nitrate was essential and this frequency range had higher sensitivity.

**Fig. 11:**
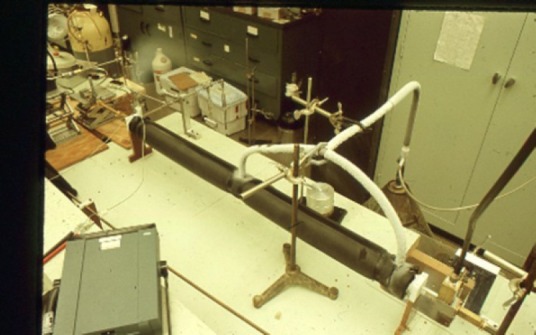
Ku-band stainless steel Stark septum cell with cooling jacket

**Fig. 12:**
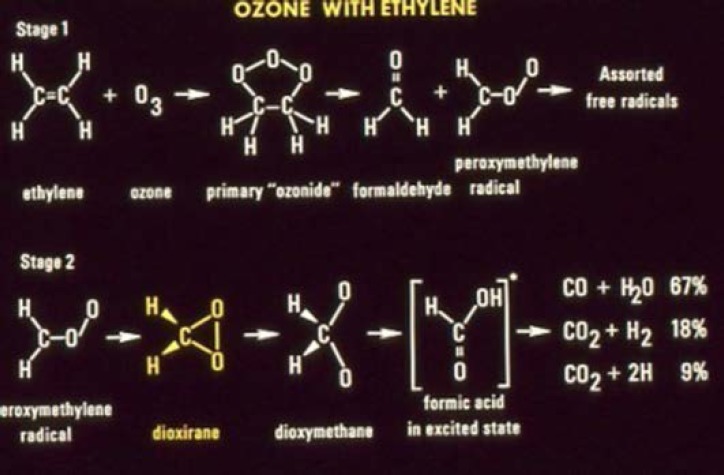
Proposed mechanism for the formation of dioxirane in the reaction of ozone with ethylene

**Fig. 13:**
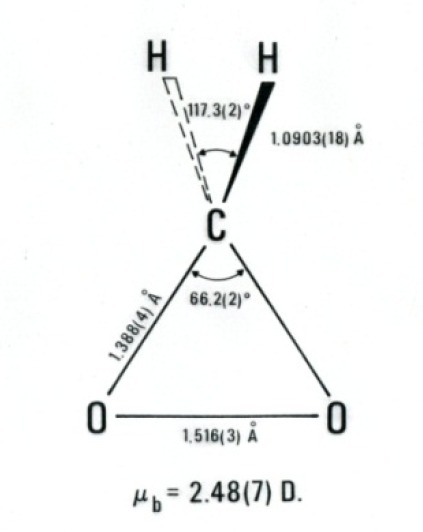
Structure of dioxirane

With some internal funding from the Office of Air and Water Measurements, Suenram and Lovas began work on the ozone-olefin reaction system, which is important in the troposphere in forming smog. Rick and Frank began a room temperature stopped flow study of ethylene and ozone reaction products in the parallel plate Stark cell. After completing a series of experiments, Rick had the idea to freeze out the two reactants in a stainless steel Stark cell, shown in Fig. 11, by introducing each separately while the cell was cooled with liquid N_2_ (preventing any reaction before warming the cell). Once the ozone and ethylene were introduced and frozen out, the cell was slowly warmed and spectral scans undertaken. Formaldehyde was observed first at a temperature of −130 °C and then the dioxirane (H_2_CO_2_) spectrum appeared between −100 °C and −84 °C before disappearing. Over the years this reaction has received considerable attention by organic chemists. The mechanism of reaction has the ozone terminal oxygen atoms adding across the C=C bond, forming a primary ozonide five-membered ring, which then cleaves to form formaldehyde and the H_2_COO radical (so called Criegee intermediate [99]) as shown in Stage 1 of the proposed mechanism for reaction pictured on the next page. Suenram and Lovas found that the H_2_COO radical is stabilized by forming the three-membered CO_2_ ring of dioxirane [100] before decomposing to the final products CO, H_2_O, CO_2_, and H_2_ measured by mass spectrometry by NBS scientists Richard Martinez, John Herron, and Robert Huie in a stopped flow study of the ozone-ethylene reaction. The proposed reaction sequence is shown in Fig. 12. It is well established that some fraction of the two initial fragments, formaldehyde and H_2_COO, recombine to form a secondary ozonide, whereby the five-membered ring has the sequence _OCOOC_ and this is also observed in the microwave experiment at temperatures above −80 °C. By employing various isotopic forms of ethylene and ozone, Suenram and Lovas studied isotopically-labeled dioxirane to obtain its substitution structure shown in Fig. 13 [101].

When Rick Suenram began his postdoctoral studies at NBS, one of his objectives was to obtain the rotational spectrum of glycine, the simplest amino acid. Once the laboratory spectrum was observed and assigned it would then be possible to search for it in the interstellar medium using radio telescopes. Unfortunately most amino acids exist at room temperature as solid substances with negligible vapor pressure. In order to get enough substance in the vapor phase for a microwave study, the amino acid has to be heated. Also by nature of being a biomolecule, amino acids are somewhat susceptible to thermal decomposition so they are difficult to vaporize without decomposing them. In order to study them Rick designed and built a millimeter wave, parallel plate, absorption cell that was capable of being heated to several hundred degrees Celsius using external heating tapes. The cell was constructed of Pyrex and gold plated metals to minimize decomposition. A small quartz boat containing the sample to be vaporized was placed in the center of the cell, directly beneath the gold plated parallel plates. Using this cell, the millimeter wave spectrum of glycine was observed and assigned [102]. As with many organic molecules, there are possibilities for conformational isomerism due to internal hydrogen bonding within the molecule. Glycine was no exception and the first conformer observed had a large dipole moment along the *a*-molecular axis. Subsequent ab-initio calculations [103] suggested that the experimentally observed conformer was in fact not the lowest energy conformer and hence might not be the most likely conformer to be observed in the interstellar medium. Nevertheless, several interstellar searches were undertaken for this conformer in collaboration with Lew Snyder and his students. These searches all turned out to be unsuccessful.

**Fig. 14:**
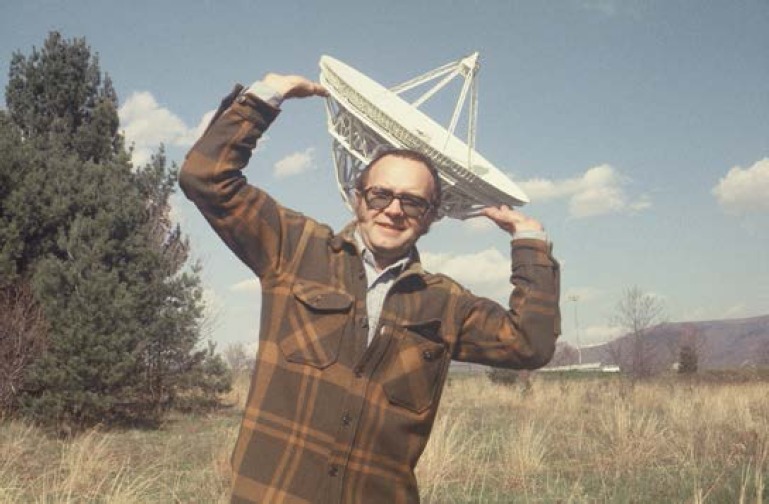
Rick Suenram assisted in pointing the NRAO 140 ft telescope at Green Bank, WV

Based on the results and predictions in Ref. [103], new laboratory searches were undertaken for the predicted lowest energy conformer of glycine. After some searching, a weaker spectrum of a second conformer was observed and assigned [104]. The spectrum of this conformer was weaker due to the fact that the dipole moment was much smaller than that for the first observed conformer. Based on the newly assigned spectrum, additional interstellar searches were undertaken but again they were unsuccessful in detecting glycine in the interstellar medium [105]. One of the NRAO telescopes employed in the interstellar searches was the 140 ft. telescope at Green Bank, WV shown in Fig. 14.

During the period 1980 to 1981 Bob Kuczkowski made a sabbatical visit to NIST working with Lovas and Suenram. One of the interesting studies Bob was involved in was the millimeter wave study of the spectrum of sulfuric acid to obtain its molecular structure. A parallel plate cell that was originally built to study glycine was used since it was all glass or gold coated metal and thus not subject to the corrosive acid. Also, it was designed to be heated to several hundred degrees Celsius by means of heating tapes. In addition to studying the normal isotopic form at temperatures near 100 °C, the ^34^S, d_1_ and d_2_ deuterated forms were studied, allowing the structure to be determined [106].

Lovas and Suenram began studies on radical and transient species employing the microwave discharge and millimeter wave parallel plate cell. Using a flowing mixture of H_2_S in N_2_, they generated the NS radical and measured the N = 1-0, 2-1 and 3-2 rotational hyperfine patterns [107] over the frequency range 67 GHz to 162 GHz and predicted the spectrum up to 300 GHz for radio astronomy applications. In addition to generating the NS radical, two new species were also identified: thiohydroxylamine (H_2_NSH) and sulfur diimide (HSNSH). Both *cis* and *trans* forms of H_2_NSH were assigned [108]. For HSNSH two of the possible three planar conformers were observed, the *cis*-*trans* and *cis*-*cis* [109].

**Fig. 15:**
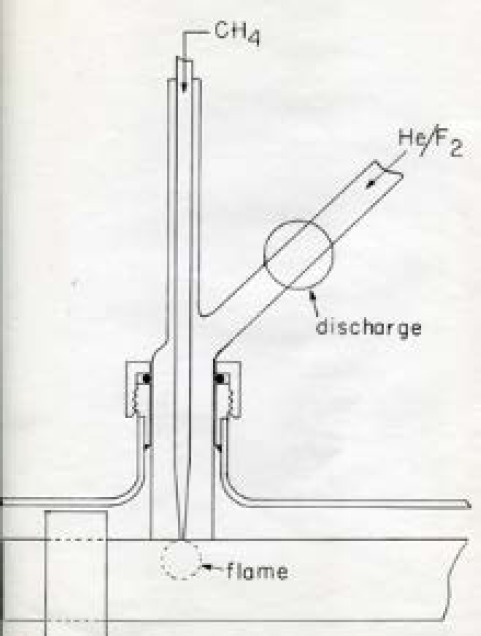
Microwave discharge setup to form CH_2_ from F atom abstraction of hydrogen from methane

In 1983, with the assistance of Ken Evenson from the NBS Boulder Lab, Lovas and Suenram began a search for the N_KK_ = 4_04_ – 3_13_ transition of the X ^3^B_1_ ground state of the CH_2_ radical between 68 GHz and 71 GHz based on predictions from the far infrared measurements from prior Evenson studies. A microwave discharge of F_2_ in He generated F atoms that were used to extract hydrogen atoms from methane as shown in Fig. 15. After some searching, three hyperfine triplets were detected corresponding to the J = 5-4, 4-3 and 3-2 transitions. This work was reported in the Astrophysical Journal due to its importance in radio astronomy [110]. While the initial search for interstellar CH_2_ was only partially successful [111], the detection was later confirmed by Hollis, Jewell, and Lovas [112] toward the Orion-KL and W51 M molecular clouds.

In the early 1980s Suenram and Lovas turned their attention to studying several species important in atmospheric chemistry, particularly in the destruction of ozone, with support from the Chemical Manufacturers Association. The first of these studies was an extension of measurements on chlorine nitrate into the millimeter wave range [98]. The second species studied was hypochlorous acid, HOCl, with collaborators from the University of British Columbia (M.C.L. Gerry’s lab) and the JPL spectroscopists Cohen and Pickett [113]. This was followed with the study of peroxynitric acid (HOONO_2_) over a frequency range from 40 GHz to 189 GHz. The spectrum of the ground state of HOONO_2_ exhibited tunneling splittings on the order of 5 MHz to 10 MHz due to tunneling of the OH across the heavy atom plane [114]. In the mid-1980’s the Molecular Spectroscopy Division initiated a new direction which focused on the spectroscopy (both infrared and microwave) of molecular dimers and clusters. Suenram and Lovas began their effort with the study of HF dimer [115] and H_2_CO-HF [116] employing the stainless-steel K_u_ band Stark septum cell previously used in the study of dioxirane. This allowed them to cool the cell to enhance the formation of dimers. Realizing that this method would not work for more weakly bound dimers, they began construction of a pulsed molecular beam Fabry-Perot cavity Fourier Transform Microwave Spectrometer (FTMW) which had recently been developed by Bill Flygare’s group at the University of Illinois (commonly known as the Balle-Flygare instrument [117].

## 5. Fabry-Perot Cavity Fourier Transform Microwave Spectrometer

**Fig. 16:**
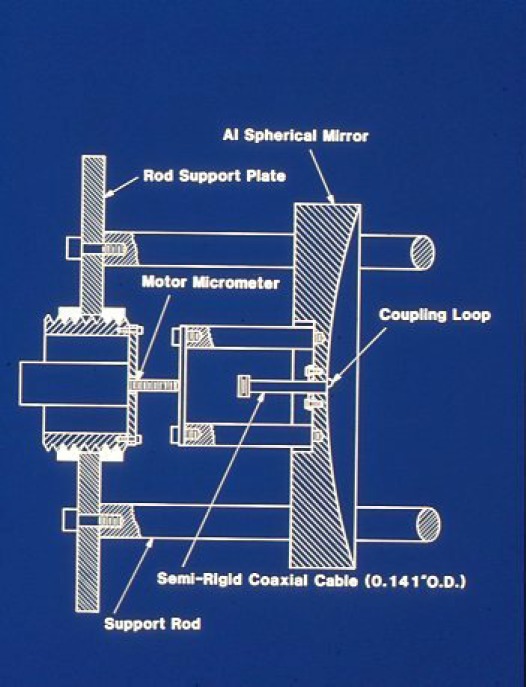
One of the Fabry-Perot resonator mirrors in the FTMW spectrometer

It turns out that this new technique would lead to a worldwide renaissance of rotational spectroscopy over the next three decades. The initial configuration of the NIST instrument was described by Lovas and Suenram in a paper on rare gas complexes of OCS [118]. This version had two microwave oscillators phase locked to each other with a 30 MHz IF and required manual stepping of the master oscillator and the Fabry-Perot cavity mirror. Mirror movement was achieved by means of manually stepping the motor micrometers behind each mirror as illustrated in Fig. 16.

Quite a large number of rare gas molecular complexes and molecular dimers were studied with this spectrometer but here we will just highlight the ones of special interest. At this point in time, the water dimer was of high interest both experimentally and theoretically due to multiple tunneling motions. Water dimer has eight equivalent frameworks giving rise to five pairs of vibration-rotation species: E^±^, A_1_^±^, B_1_^±^, A_2_^±^, and B_2_^±^. The initial lab study at NBS was reported in Ref. [119] and later Coudert and Hougen reported the theoretical model and a global analysis of all microwave and far infrared data available [120]. This included the data reported by Fraser, Suenram, and Coudert taken with the newly developed microwave electric-resonance optothermal spectrometer [121].

**Fig. 17:**
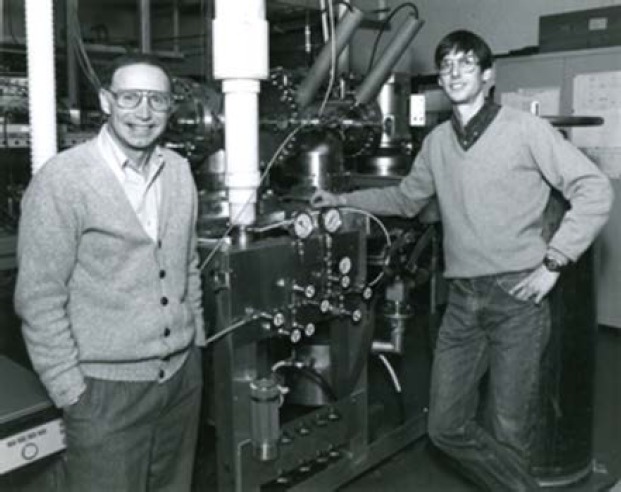
Alan Pine (left) and Gerald Fraser next to the microwave and infrared electric-resonance optothermal spectrometer (EROS) about 1989

The ammonia dimer is another example of a species exhibiting complex tunneling motions. Jerry Fraser, who came to NBS as a Postdoctoral fellow working with Alan Pine (see Fig. 17), had initially studied the ammonia dimer with the molecular beam electric resonance spectrometer at Harvard with Nelson and Klemperer. Jerry carried out zero field hyperfine measurements of four isotopic variants of ammonia dimer with the FTMW spectrometer [122].

During 1987–88 Woody Gillies and Jennifer Zozom (Gillies) made several extended visits to the NBS microwave lab. They participated in the FTMW study of the formamide–water and formamide-methanol complexes [123]. Both complexes were found to have two hydrogen bonds, the oxygen of water (methanol) bound to the H of the HNCO moiety and the OH hydrogen to the oxygen atom of the HNCO group.

**Fig. 18:**
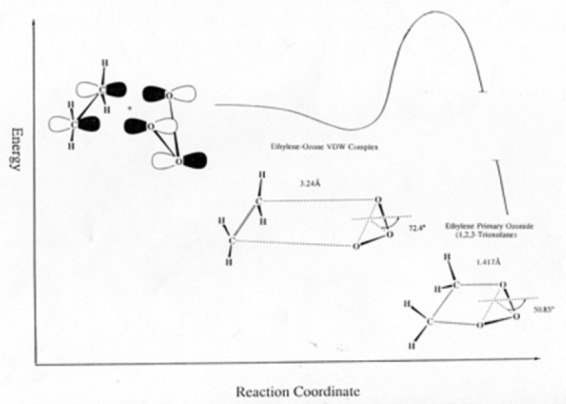
Reaction coordinate for ethylene plus ozone in forming the ethylene primary ozonide

During one of these visits they also returned to studies of the ozone-ethylene system in an attempt to produce the primary ozonide of ethylene. This is the five-member ring structure where the terminal O-atoms of ozone bridge the C–C bond and produces the initial product in the reaction scheme shown earlier. However, due to its instability, no one had ever observed it spectroscopically. The same experimental procedure used in the dioxirane study was followed, except the cell temperature was maintained near −100 °C for up to 6 hours after warm up from −196 °C by flowing cooled N_2_ gas through the cylinder surrounding the waveguide cell. By this method, the primary ozonide of ethylene was stabilized and its millimeter wave spectrum was observed [124]. Later, a structural study was carried out using various isotopic forms enriched in ^13^C, D, and ^18^O [125]. A year later, on another visit by the Gillies, the ozone-ethylene van der Waals complex was studied with the FTMW spectrometer [126]. The complex was found to have the same basic structure as the primary ozonide defining the reaction coordinate of the 1,3-dipolar cycloaddition leading to the primary ozonide shown in Fig. 18. A more detailed structural study was reported several years later [127].

**Fig. 19:**
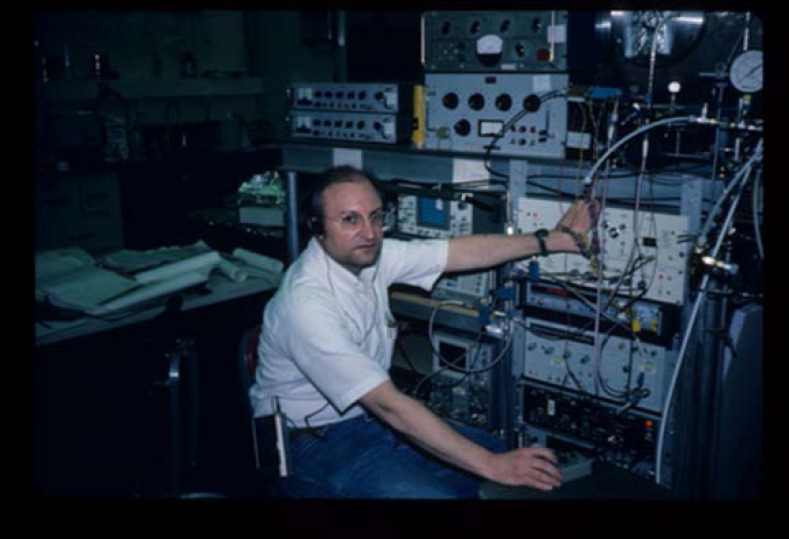
Stewart Novick operating the NBS FTMW spectrometer

In 1988 Stew Novick, shown in Fig. 19, visited NBS on sabbatical from Wesleyan University. His interest was the structural study of the van der Waals heterodimer OCS-CO_2_. It was anticipated that the dimer would have a slipped parallel structure like the carbon dioxide dimer, and this proved to be the case [128]. Later on Stew duplicated one of the NBS FTMW spectrometers for his use at Wesleyan based on the drawings provided by Suenram and Lovas. Later the Wesleyan shop constructed three others for use at Harvard, Mt. Holyoke College, and Amherst College.

About this same time Helen Leung and Mark Marshall, came to NBS to study the N_2_-H_2_O complex which exhibits the same type of interchange of the donor and acceptor atoms in the H-bond as does water dimer, however only four equivalent frameworks exist in this case [129]. During 1988 and later Karen Peterson from the University of Rhode Island came to NBS with an interest in studying trimers containing water. The first study involved CO_2_ and H_2_O to form the CO_2_-CO_2_-H_2_O complex [130]. The structure found contained the planar CO_2_ dimer in a slipped parallel structure with the water above the plane and the water oxygen lone pair directed at the two carbon atoms. No tunneling was detected. The second study also involved CO_2_ and H_2_O but the trimer complex formed was H_2_O-H_2_O-CO_2_. The structure found has all the heavy atoms in a plane and perhaps the hydrogens as well since the dipole moment in the c-axis direction is zero [131].

**Fig. 20:**
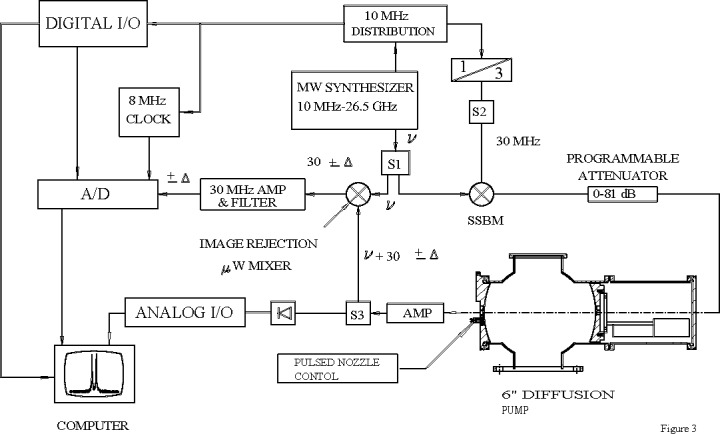
Schematic diagram of the portable FTMW spectrometer developed at NIST in the late 1990’s

In all the studies mentioned to date, the FTMW instrument was manually scanned and this was a major impediment to getting things accomplished in a timely fashion. It was time to move on to the next step in instrument development and improvement. In order to automate the scans, a number of electronic modifications to the FTMW instrument were first necessary. A single-sideband modulator (SSBM) was introduced in the electronics along with a 30 MHz source (tripling the 10 MHz reference signal), which allows one to remove the second microwave oscillator by using a power splitter with one output to the SSBM and the second output to the detector mixer [132]. The schematic in Fig. 20 shows the layout that was ultimately used in the development and construction of the portable FTMW spectrometers at NIST in the mid to late 1990s [133]. With all the improvements that were made during approximately a decade of experiments, the sensitivity of the FTMW instrument was now in the ppb range making it viable to observe many isotopomers in natural abundance.

**Fig. 21:**
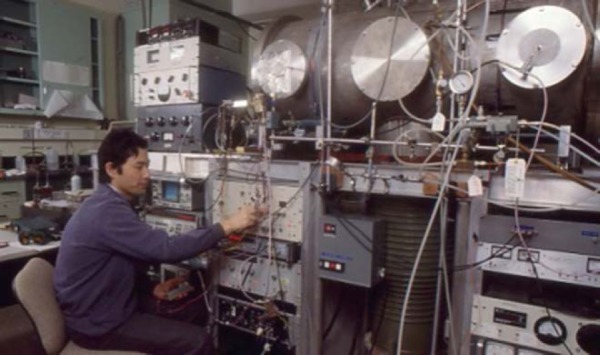
Keiji Matsumura using the original FTMW spectrometer

Keiji Matsumura from Seinan Gakuin University in Japan, shown in Fig. 21, spent a sabbatical year working with Lovas and Suenram on laser ablation studies as well as molecular dimers. One of the dimer studies Keiji carried out was on the deuterated and partially deuterated acetylene dimer [134] which exhibits interesting tunneling.

**Fig. 22:**
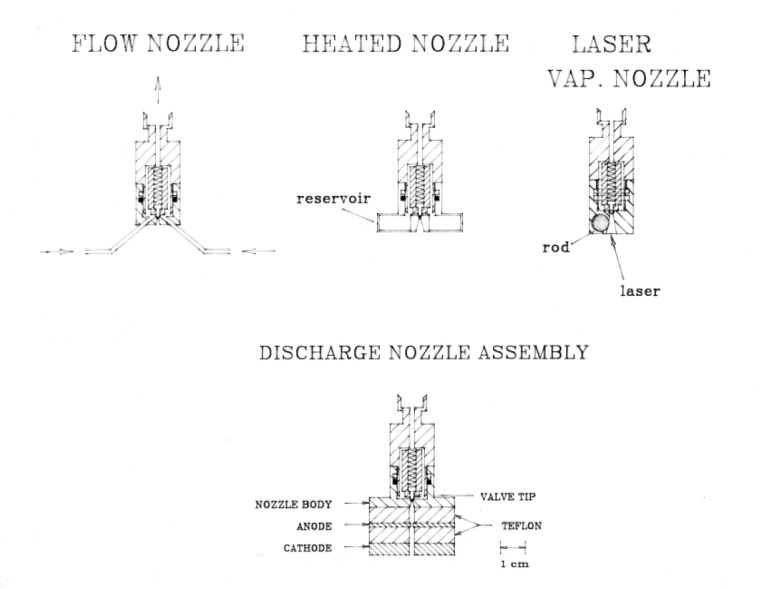
Several pulsed nozzle designs developed by Suenram and Lovas for the FTMW spectrometer

As the FTMW technique began to mature in the late 1980s and early 1990s, Suenram and Lovas looked to ways to extend the technique beyond simple hydrogen bonded species and van der Waals clusters. One of the most fruitful means was to employ different nozzle designs. Suenram and Lovas developed a variety of pulsed nozzle configurations to facilitate the study of other types of molecular compounds and complexes. Several of the nozzles designs that they developed are shown in Fig. 22.

The flow nozzle was employed when the two species whose complex was sought reacted and formed a solid, e.g. NH_3_ and HX (X = halogen). The flow nozzle was also used in the ozone-ethylene experiments to observe the ozone-ethylene van der Waals complex [126]. During several more visits by Woodie and Jennifer Gillies, the dual flow nozzle also proved useful in studying ketene-acetylene [135] and ketene-ethylene [136]. These were of interest because Woodward and Hoffmann suggested that the reaction could be viewed as a concerted (2π_s_ + 2π_a_) cycloaddition with a transition state characterized by crossed, mutually perpendicular molecular planes of ketene and the alkene. In the case of ethylene the structure found was close to what Woodward and Hoffmann suggested, but for acetylene a planar structure was found.

**Fig. 23:**
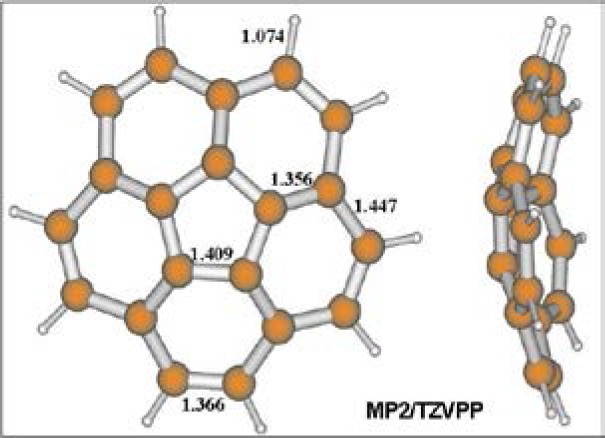
*Ab initio* structure of corannulene

The reservoir nozzle can be heated routinely up to 200 °C for species with low vapor pressure at room temperature. It has been employed extensively over the years at NIST and the design has been reproduced and used at many other microwave labs around the world. Probably one of the most difficult experiments using this nozzle involved the study of corannulene (C_20_H_10_) where Lovas and Grabow heated the nozzle to 220 °C to 250 °C [137]. In the course of this study, several nozzle coils burned out due to the high temperature employed. Corannulene, with the structure in Fig. 23, was of interest since it is polar, as opposed to many of the polycyclic aromatic hydrocarbons (PAHs) which are non-polar and have no microwave spectrum. The PAHs are thought to be the carriers of the interstellar diffuse bands.

**Fig. 24:**
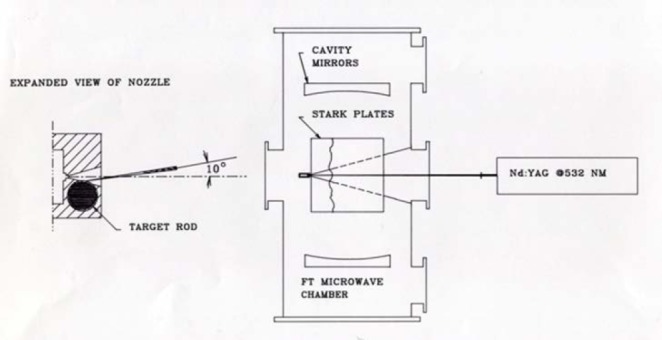
Configuration used for laser vaporization of solid materials

The DC discharge nozzle was employed in the study of radicals or transient interstellar species to measure low-J rotational transitions and to determine the dipole moments [138,139]. The laser ablation pulsed nozzle source was used to study refractory materials or other solids with low vapor pressure. Some of the first studies involved SiC_2_ [140] and the refractory metal oxides YO, LaO, ZrO, and HfO [141]. Later this technique was used for microwave measurements on BaO and SrO and combined with new infrared measurements on SrO and earlier infrared measurements to give improved Dunham potential constants for BaO and SrO [142]. These experiments were the first applications coupling laser vaporization to an FTMW instrument. It also showed that the FTMW instrument had the sensitivity to observe refractory compounds. Note that the oxides of Y, La, Zr, and HF were previously only seen using optical absorption experiments. The diagram in Fig. 24 shows the basic configuration employed.

In the early 1990s the long-standing relationship with the Russian millimeter wave group headed by A. F. Krupnov was greatly strengthened. This was made possible in part by the lifting of the iron curtain. Rick made two trips to Nizhny Novgorod (formerly Gorky) in the early 1990s to work firsthand with many members of Krupnov’s group [143]. Prior to this, Nizhny Novgorod was a closed city, inaccessible to Westerners. In this same timeframe, NIST also hosted a number of Krupnov’s staff where Lovas and Suenram worked on many projects of mutual interest both in the conventional millimeter frequency region and the FTMW region. Among those that visited NIST were S. P. Belov, M. Yu. Tretyakov, and E. N. Karyakin. Belov and Tretyakov worked with Lovas and Suenram on the study of several molecular complexes, namely CH_3_OH-CO [144] and methanol dimer [145]. One of the interesting results of these studies was that the internal rotation barrier of the CH_3_ rotor was substantially lowered, a factor of two or more compared to free methanol. Another interesting aspect of the methanol dimer study was that 16 transitions occur for each of the K = 0, a-type rotational transitions. One of the early studies that Karyakin also carried out was the donor-acceptor tunneling in HDO-DOH and HDO-HOD [146].

**Fig. 25:**
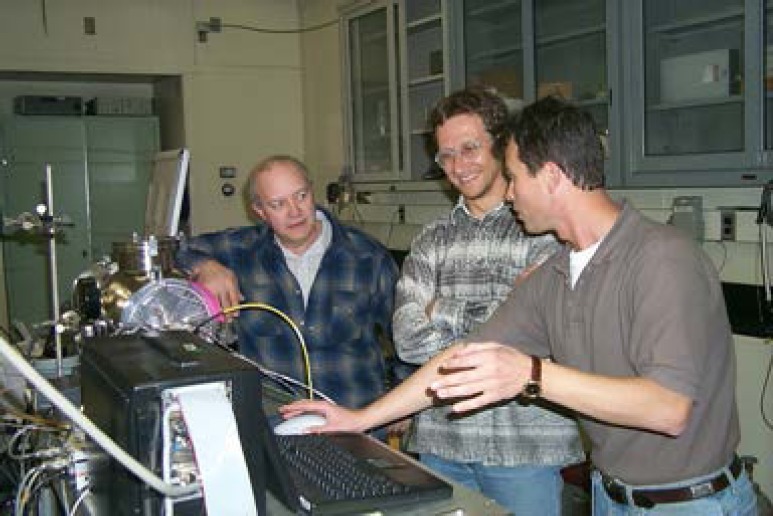
Rick Suenram (left) with Andre Zuban (middle) and Igor Leonov (right)

Of particular note is the work with Andrey Zuban and Igor Leonov, shown in Fig. 25 with Rick Suenram, on the automation of the FTMW spectrometers at NIST. Andrey and Igor are hardware and software specialists, respectively, who had more than a thorough understanding of using computers to automate instrumentation. Together they developed a software package known as LZ98 that was used to control FTMW spectrometers, not only at NIST but also at other labs in the US, Europe, and Japan [133]. This software permitted long (many GHz), unattended searches to be carried out with this type of spectrometer thus making it possible to set up a particular set of chemistry conditions and blindly search to see what was present in the chemical system being studied without having any preconceived notions of where to look for a particular species. It cannot be emphasized strongly enough how important this was in every type of system studied by FTMW instruments all the way from large organic compounds where multiple conformers are present (1-octene, 15 conformers [147]) to small metal oxides produced by laser vaporization (ZrO_2_ [148], HfO_2_ [149]). In the metal oxide experiments, a number of strong transitions were observed in the several GHz that were scanned. It turned out that these transitions belonged to the metal dioxides, which had never been seen previously in the gas phase.

Suenram and Lovas entered a major long-term collaboration with the rotational spectroscopist, Jens-Uwe Grabow, from Kiel, Germany. In the mid 1990s Jens began the first of several extended visits to NIST as a Postdoctoral fellow. One of the most noteworthy studies Jens carried out at NIST was on the very weakly bound rare gas dimer Ne-Ar which required a 1 Watt amplifier to boost the microwave input power to the cavity [150]. Jens and Yoshi Kawashima, another frequent visitor to the NIST lab, also worked on the spectrum of conformers I and II of glycine to determine the dipole moments and resolve the ^14^N hyperfine structure. In these studies, glycine was produced both in a heated nozzle and by laser vaporization [151]. Jens was also principal author on the FTMW study of N_2_O_5_ which exhibited large amplitude motions from the two NO_2_ groups which undergo internal rotation tunneling via a geared rotation of the two units about their C_2_ axes [152].

Jens also developed an automated software package for control of the FTMW type instruments but one of the main collaborations at NIST was working with Suenram on the development of a scaled down (smaller) version of the FTMW instrument that was totally transportable [133]. In the late 1990s, several of these spectrometers were constructed. One of these instruments was taken to Aberdeen, Maryland and installed in an Army surety laboratory and used to observe and analyze the rotational spectra of several of the nerve agents for the first time, first Sarin [153], and then Soman [154]. This instrument and one of the sister instruments at NIST were also used to study a large number of agent related compounds from 2000 to 2005 with support from the Army Research Office [154,155]. Another study by Suenram and coworkers was on dimethyl methylphosphonate which is among the organo-phosphorus compounds which are relatively harmless but serve as model compounds in place of nerve agents. The spectrum showed an unexpected complication in that the methoxy groups tunneled to produce equivalent structures in addition to the internal rotation of the three methyl groups [156]. In the initial report only the A-state was fit, but later a global analysis was carried out [157].

In the mid-1990s, David Plusquellic was hired at NIST. David’s area of expertise is high resolution UV, visible laser spectroscopy but he has had a major impact in microwave spectral analysis. During his tenure as a graduate student at David Pratt’s laboratory at the University of Pittsburgh he began the development of a Graphical User Interface (GUI) program to simulate optical spectra. The program is known as JB95 and is available from NIST at http://www.nist.gov/pml/div682/grp01/jb95.cfm. One advantage of this program was that it utilized the ground state rotational constants to simulate rotational spectra. One could overlay a theoretical spectrum from assumed or *ab initio* calculated rotational constants on the laboratory scan. The assumed rotational constants, dipole moment components, and resulting spectra are all computer mouse controlled by track bars. This permits rotational assignments to be varied “on the fly” to see if a match can be obtained to the observed spectrum (spectra). Once a close match or pattern is observed, the transitions can be assigned in a “point and click” fashion using a computer mouse and trial fits obtained. This literally reduced assignment times to sometimes a few minutes for spectra that might otherwise have taken days or weeks to analyze. Using this program, one can simulate up to nine separate spectra at once. Also, once a particular spectrum (conformer) has been assigned, it can be digitally subtracted from the overall spectrum to simplify the remaining spectra. This is true not only for multiple conformers, but isotopomers as well (^13^C species for instance). Needless to say, it would have been difficult if not impossible to study and assign the spectra of quite a few of the larger molecules that we have studied without the use of this program, e.g. see 1-pentene [158], 1-octene [147], and 1-heptanal [159] for instance.

**Fig. 26:**
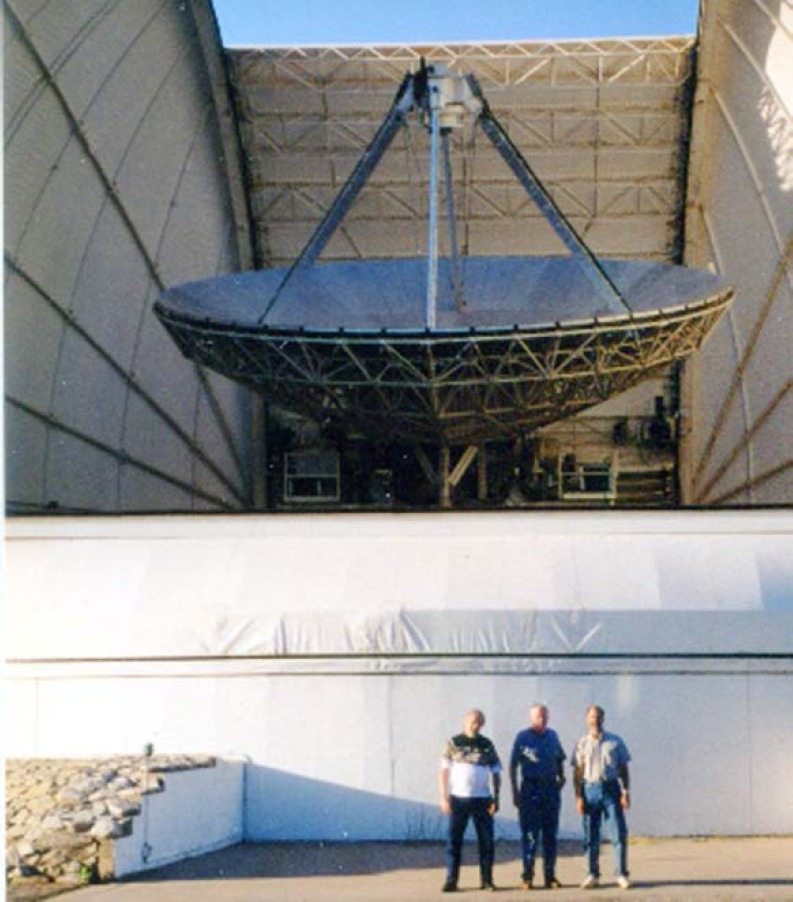
Lovas, Hollis and Jewell at the NRAO 12 m in May 2000

In early 2000 Lovas’ interest returned to interstellar molecules. In May 2000 Hollis, Lovas, and Jewell used the NRAO 12 m radio telescope near Tucson, AZ (shown in Fig. 26) to study the simplest sugar, glycolaldehyde. They detected 6 transitions in the galactic center cloud Sgr B2(N) at frequencies between 71 GHz and 104 GHz [160]. This was the last time the group used the 12 m, since in June 2000 NRAO turned over the operation of the instrument to the Astronomy Department of the University of Arizona.

In a later study of interstellar glycolaldehyde with the BIMA array telescope, Hollis et al. [161] showed that the spatial extent exceeded 60′ rather than being confined to the hot core (about 5′ in diameter) of the cloud Sgr B2(N-LMH). From the same data taken in May 2000, Hollis and co-workers identified ethylene glycol (antifreeze), which is the sugar alcohol of glycolaldehyde [162]. Shortly after the glycolaldehyde detection, Lovas spent six months at the Astronomy Department of the University of Illinois working with Lew Snyder and his students.

Meanwhile at NIST, Fraser, Suenram and a visiting scientist Catherine Lugez embarked upon a series of studies involving 1-alkenes which were known to have large numbers of molecular conformers in room temperature gas phase samples. It was surmised that in a molecular beam, a much simpler picture would emerge as all the higher energy conformers would cool down to the lowest energy conformer and only the spectrum of the lowest energy conformer would be seen in the molecular beam. The authors were surprised by the results in that the spectrum that was observed was quite complex, indicating that a number of conformers were present in the molecular beam. While it is true in some cases that conformers will freeze out, if there are barriers to internal rotation that inhibit a higher energy conformer from getting to a lower energy conformer, the individual conformers will remain suspended in the gas phase and emerge from the nozzle unscathed. (While the alkanes were of primary interest, they tend to have very small dipole moments and thus are not amenable to microwave studies.) The C=C double bond in the alkenes assures that all have a dipole moment. The first study reported on seven conformational isomers of the expected 13 for 1-hexene [158]. This was followed with the observation of four of the five expected conformers of 1-pentene [163]. A year later these authors reported the observation of fifteen conformers of 1-octene [147].

**Fig. 27:**
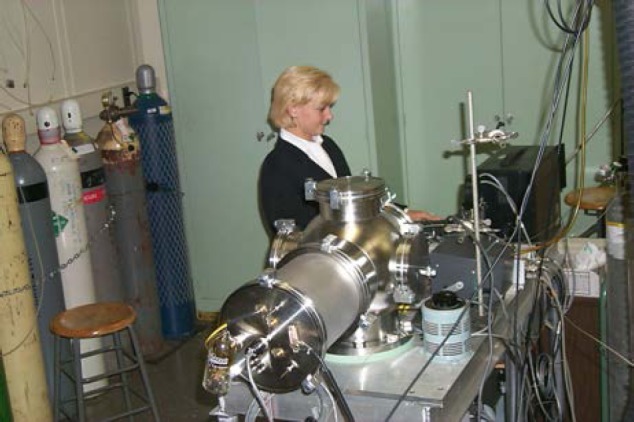
Angela Hight Walker shown with one of the portable FTMW spectrometers at NIST

While the study of molecular complexes was waning at NIST, the ozone-methane complex drew the interest of Angela Hight Walker, shown in Fig. 27, and coworkers since it was involved in the study of the O + CH_4_ reaction as a means to fix the time zero in measuring the product appearance in this fast reaction. Thus the geometry and internal dynamics of this complex was of interest. The FTMW study of the O_3_-CH_4_ complex showed that the CH_4_ undergoes nearly free internal rotation which leads to A, E, and F internal-rotation sublevels which correlate to those three rotational states in free methane [164].

Rich Lavrich joined the group as a postdoctoral fellow from 2001 to 2003. He initially worked with Suenram, but when Suenram retired Lavrich was supervised by David Plusquellic. Together they carried out a number of studies on conformational analysis of peptide mimetics. The first of these was on the alanine dipeptide analog *N*-acetyl-alanine *N*′-methylamide [165]. With three methyl rotors, 14 torsional states are expected. However only three of these were analyzed, the AA, AE, and EA states which accounted for the vast majority of the spectral features observed. A second study Rich carried out was on the peptide mimetic ethylacetamidoacetate (also known as *N*-acetylglycine ethyl ester) that yielded two low energy conformers [166].

**Fig. 28:**
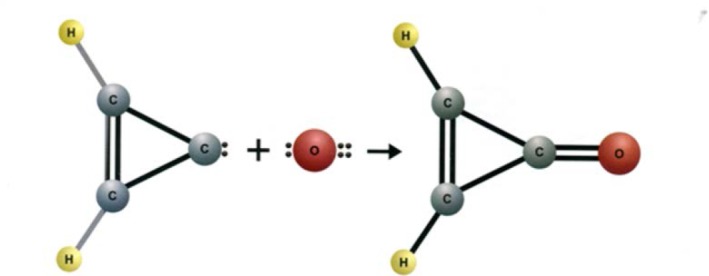
Proposed formation route to produce interstellar cyclopropenylidene

The Green Bank Telescope (GBT) was found to be very good for the detection of larger organic molecules, e.g. the aldehydes propenal and propanal [167]. Lovas and co-workers identified cyanoallene toward the TMC-1 molecular cloud aided by its hyperfine structure [168]. This detection was followed by the detection of a new ring molecule, cyclopropentone (c-H_2_C_3_O) also with the 100 m GBT [169]. The authors suggested a formation route for cyclopropenone via the addition of an oxygen atom to cyclopropenylidine (c-C_3_H_2_) as shown in Fig. 28.

**Fig. 29:**
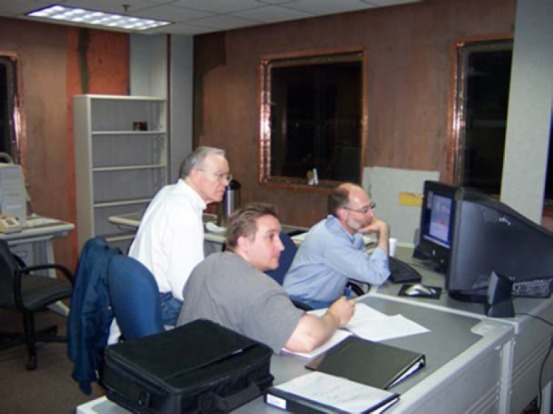
Hollis, Remijan and Jewell in the GBT control room

**Fig. 30:**
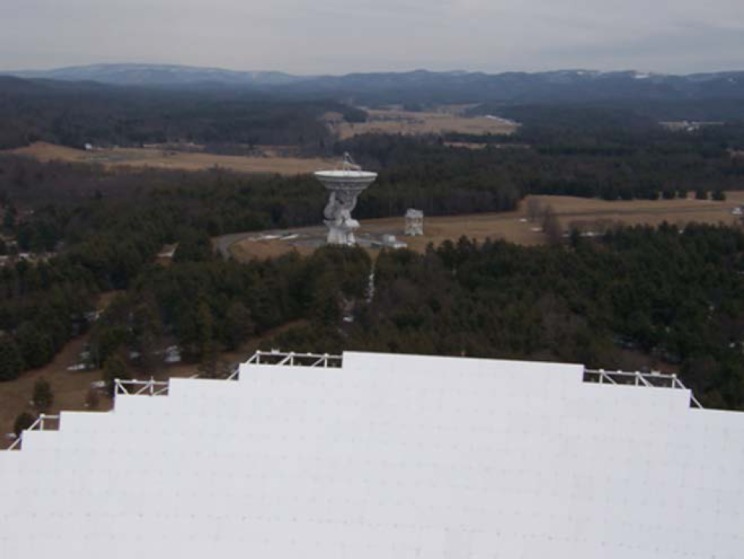
View of the NRAO 140 ft from the top of the GBT

Shortly after this methyltriacetylene (CH_3_C_6_H) was detected toward TMC-1 [170]. This is the largest symmetric top molecule detected to date. Working from GBT data collected in 2004 and 2005, Hollis and coworkers identified interstellar acetamide (CH_3_C(=O)NH_2_) toward Sgr B2(N-LMH) by means of 7 A and E state rotational transitions [171]. This is the largest interstellar molecule with a peptide linkage, i.e. the HNCO backbone. These workers reported the detection of keteneimine (CH_2_=C=NH) toward the hot core of Sgr B2(N) which is a higher energy isomer of methyl cyanide [172]. In Fig. 29 three members of the observing team, Hollis, Remijan, and Jewell are shown in the GBT control room. Figure 30 shows a view of the NRAO 140 ft. telescope form thje top of the GBT receiver room.

The last two parts of the series “Microwave Spectra of Molecules of Astrophysical Interest” were published in 2007 and 2008. These were Part XXV on methylamine [173] and Part XXVI on acetic acid [174], two important interstellar species exhibiting internal rotation. In March of 2008 Remijan and coworkers reported the detection of cyanoformaldehyde toward Sgr B2(N) with the GBT [175]. The formation mechanism is postulated to be from a radical neutral molecule reaction of CN with H_2_CO yielding CNCHO + H. The team of Hollis, Lovas, Jewell and Remijan detected 8 new interstellar species with the GBT over a two year period.

Returning to laboratory studies, several interesting polyols with three carbon atoms were studied as potential interstellar molecules. The first of these was glycerol, CH_2_OHCHOHCH_2_OH, which has 12 conformational isomers. From the FTMW spectral scans 5 conformers were identified with the aid of *ab initio* calculations. One of these showed an interesting tunneling between its two chiral forms [176]. The second study was on 1,2-propanediol. It is an asymmetric top molecule with at least 23 low energy conformers. Its spectrum was scanned with the NIST cavity FTMW spectrometer as well as the broadband chirped-pulsed FTMW spectrometer in Brooks Pate’s lab at the University of Virginia. The seven lowest energy conformers were assigned with the aid of *ab initio* calculations. The two lowest energy forms were sought unsuccessfully with the GBT toward Sgr B2(N-LMH) [177]. We thought this might be successful since it only differs from the known interstellar molecule ethylene glycol by an additional methyl group. The final study of these C_3_ polyols was on 1,3-propanediol. As in the previous case both cavity and broadband FTMW spectrometers were used. *Ab initio* calculations were carried out on the 8 lowest energy forms to aid the spectral analysis. In this case only the two lowest energy conformers were assigned and both exhibited tunneling between their two chiral forms (mirror images) [178].

**Fig. 31:**
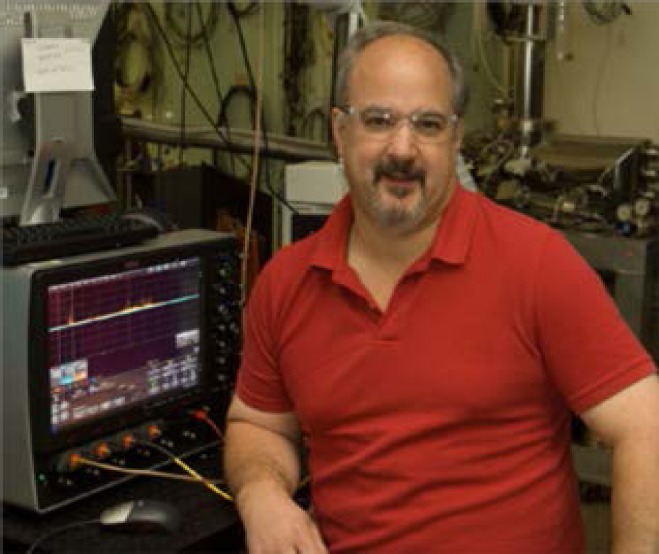
David Plusquellic with the broadband terahertz spectrometer

**Fig. 32:**
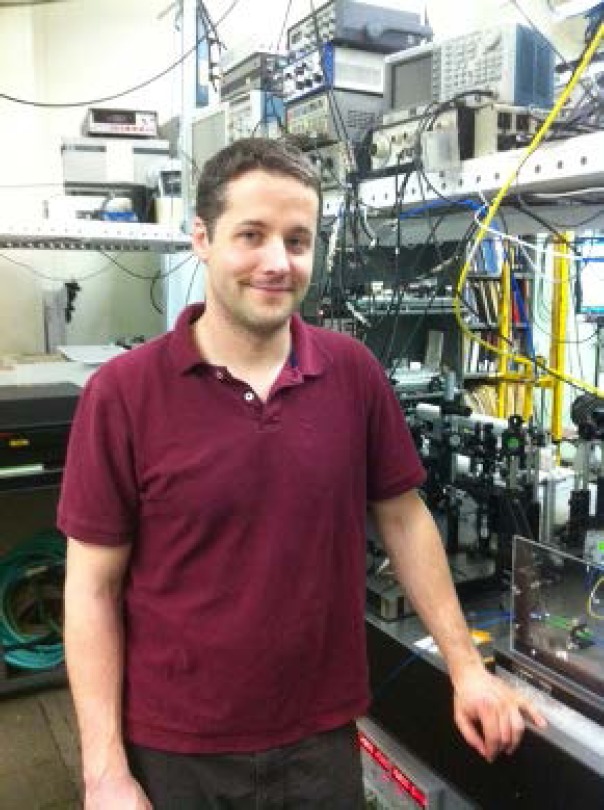
Kevin Douglass

Both Frank Lovas and Rick Suenram formally retired from NIST in 2000 but remained as guest scientists for a number of years. At the moment only Lovas remains in the microwave lab, so before long this story will end for the microwave lab. However, Dave Plusquellic (Fig 31) and Kevin Douglass (Fig. 32) have recently developed a chirped-pulse broadband terahertz spectrometer in the region (300 GHz to 900 GHz) and demonstrated its sensitivity for trace gas sensing [179]. Thus, future rotational spectroscopy at NIST will likely move to their laboratory.

In the references and cited in the text, nearly all of the lab studies reported up to about 1970 are given. After 1970, because of space limitations, we have only described roughly 25 % of those published during this very active period. For a more complete list the reader is encouraged to search the Web of Science (or similar databases) for publications from individual staff members and their visitors.
